# Prediction of fluid responsiveness in spontaneously breathing patients with hemodynamic stability: a prospective repeated-measures study

**DOI:** 10.1038/s41598-024-65554-8

**Published:** 2024-06-24

**Authors:** Yong Hwan Kim, Jae Hoon Lee

**Affiliations:** 1 Department of Emergency Medicine, Samsung Changwon Hospital, Sungkyunkwan University School of Medicine, 158 Palyong-ro, Masanhoiwon-gu, Changwon-si, Gyeongsangnam-do 51353 South Korea; 2 Department of Emergency Medicine, Dong-A University College of Medicine, 26 Daesin Gonwon-Ro, Seo-Gu, Busan, 49201 South Korea

**Keywords:** Hypovolemia, Stroke volume, Echocardiography, Hemodynamic parameters, Posture changes, Cardiology, Medical research

## Abstract

Evaluating fluid responsiveness with dynamic parameters is recommended for fluid management. However, in hemodynamically stable patients who are breathing spontaneously, accurately measuring stroke volume variation via echocardiography and passive leg raising is challenging due to subtle SV changes. This study aimed to identify normal SV changes in healthy volunteers and evaluate the precision of hemodynamic parameters in screening mild hypovolemia in patients. This prospective, repeated-measures, cross-sectional study screened 269 subjects via echocardiography. Initially, 45 healthy volunteers underwent a fluid challenge test, the outcomes of which served as criteria to screen 215 ICU patients. Among these patients, 53 underwent additional fluid challenge testing. Hemodynamic parameters, including medians of maximum velocity time integrals (VTImaxs), peak velocity of VTImax (PV), internal jugular vein diameters (IJVD), and area (IJVA) were repeatedly measured first at a 60° upper body elevation (UBE), second in a supine position, third at UBE, fourth in a supine position, and lastly in a supine position after fluid loading. The hemodynamic responses to the position changes were compared between 83 fluid non-responders and 15 fluid responders. Fluid responsiveness was defined as fluid-induced medians’ change of VTImaxs (fluid-induced median VTImax change) ≥ 10%. None of the healthy volunteers showed the mean value of repeatedly measured medians of VTImaxs ≥ 7%, following either UBE position (UBE-induced median VTImax change) or fluid loading (fluid-induced median VTImax change). UBE-induced median VTImax and PV changes were significantly correlated with fluid responsiveness (*p* < 0.001, AUC 0.959; *p* < 0.001, AUC 0.804). The significant correlations were demonstrated via multivariable analysis using binary logistic regression (*p* = 0.001, OR 90.1) and the correlation coefficient (R^2^ = 0.793) using linear regression analysis. UBE-induced median VTImax changes (≥ 11.8% and 7.98%) predicted fluid-induced median VTImax changes ≥ 10% and 7% (AUC 0.959 and 0.939). The collapsibility and variation of IJVD and IJVA showed no significant correlation. An increase in the mean value of medians of repeatedly measured VTImaxs transitioning from an UBE to a supine position, effectively screened mild hypovolemia and demonstrated a significant correlation with fluid responsiveness in spontaneously breathing patients maintaining hemodynamic stability.

## Introduction

In hemodynamic assessment, real-time monitoring of stroke volume variation (SVV) and vascular changes through ultrasound imaging has become increasingly valuable. Fluid responsiveness is often evaluated using passive leg raising (PLR) maneuvers. Among various methods, SVV is recognized as the most precise^1^. Additionally, internal jugular vein (IJV) collapsibility has gained attention, with studies suggesting their potential as more reliable indicators of fluid responsiveness compared to inferior vena cava (IVC) changes^2^. In spontaneously breathing patients, variations in IVC are affected by breathing patterns^3^. Given the substantial influence of intra-abdominal pressure on the IVC, monitoring the IJV might be more informative^4,5^. These observations necessitate further investigation into whether SV and IJV changes, coupled with PLR assessment, could provide a practical approach for evaluating volume status in spontaneous breathing patients.

SVV derived from PLR has been shown to accurately assess fluid responsiveness in both mechanically ventilated and spontaneously breathing patients^1,6^. Nonetheless, this approach faces challenges, especially in spontaneously breathing patients who are hemodynamically stable. First, the hemodynamic effect of isolated leg elevation (upper body elevation is excluded from PLR) is temporary and gradually diminishes. The maximum effect of PLR is typically achieved within 1 min^7^, lasting between 2 and 10 minutes^8^. Intriguingly, isolated leg raising manifests its hemodynamic effects within 3 min, which dissipates after 7 min, and has no impact on SV when performed after maintaining the supine position for 45 min^9^. Comparatively, changes in SV measured after isolated leg raising from a supine position were around 3%, while changes in SV after transitioning from a supine to a semi-recumbent position were 21.9%^10^. The hemodynamic effect of isolated leg raising is notably less pronounced when compared to that of upper body elevation. Second, the accuracy of SV measurements is compromised in spontaneously breathing patients. In these cohorts, cardiac output (CO) quantification through thermodilution methods showed suboptimal accuracy^11^. Maintaining regular and consistent breathing patterns may be crucial for assessing fluid responsiveness in these patients^12,13^. Third, the criteria for assessing fluid responsiveness are not well-defined. Fluid responsiveness has been defined as a ≥ 10–15% increase in CO following a 500 mL bolus fluid challenge. The thresholds of parameters such as pulse pressure variation and SVV exist in a grey zone, ranging between 10 and 15%. Changes in tidal volume, chest wall compliance, myocardial contractility, arterial tone, and intrathoracic and intra-abdominal pressures may influence SVV^14^. Lastly, while the utility of PLR-derived SVV has been mainly established in hemodynamically unstable patients, its efficacy in stable patients is yet to be confirmed^1^.

To enhance SV assessment accuracy, our study focused on higher upper body elevation, excluding the variable of leg raising and measurement methods of SV. Addressing the inaccuracies inherent in SV measurements due to spontaneous respiration, we conducted repeated measurements at the same position and utilized refined techniques, such as median values of maximum velocity time integral values (median VTImax), which were repeatedly measured during end-expiratory respiration. The primary objective of this study was to clarify the uncertain thresholds of fluid responsiveness by examining SV changes in healthy and euvolemic volunteers, and to distinguish fluid responsiveness in hemodynamically stable patients. Additionally, the study aimed to determine the predictive value of position-induced IJV changes for fluid responsiveness.

## Methods

### Study design and setting

This repeated-measures, cross-sectional study was conducted from March 1, 2020, to Dec 31, 2023, aiming to discern fluid responsiveness in hemodynamically stable subjects. Diverging from previous studies that primarily focused on hemodynamically unstable patients, our research concentrated on stable subjects due to the lack of evidence in this population. The Dong-A university hospital institutional review approved the study protocol (DAUHIRB-19-211) on August 23, 2019. All experiments were performed in accordance with relevant guidelines and regulations. Written informed consent was obtained from all subjects or their caregivers before the ultrasonographic evaluation. VTImax and other hemodynamic parameters, including mean arterial pressure (MAP), IJV diameter, and area, were repeatedly measured: first at a 60° upper body elevation (UBE), second in a supine position, third at UBE, fourth in a supine position, and lastly in a supine position after fluid loading. Measurements were taken 1 min after changing the subjects’ position. Changes in hemodynamic parameters in UBE and supine positions were investigated for predicting and assessing fluid responsiveness defined as fluid-induced medians’ change of VTImaxs ≥ 10%.

### Manipulation and the evidences

**Figure 1:**
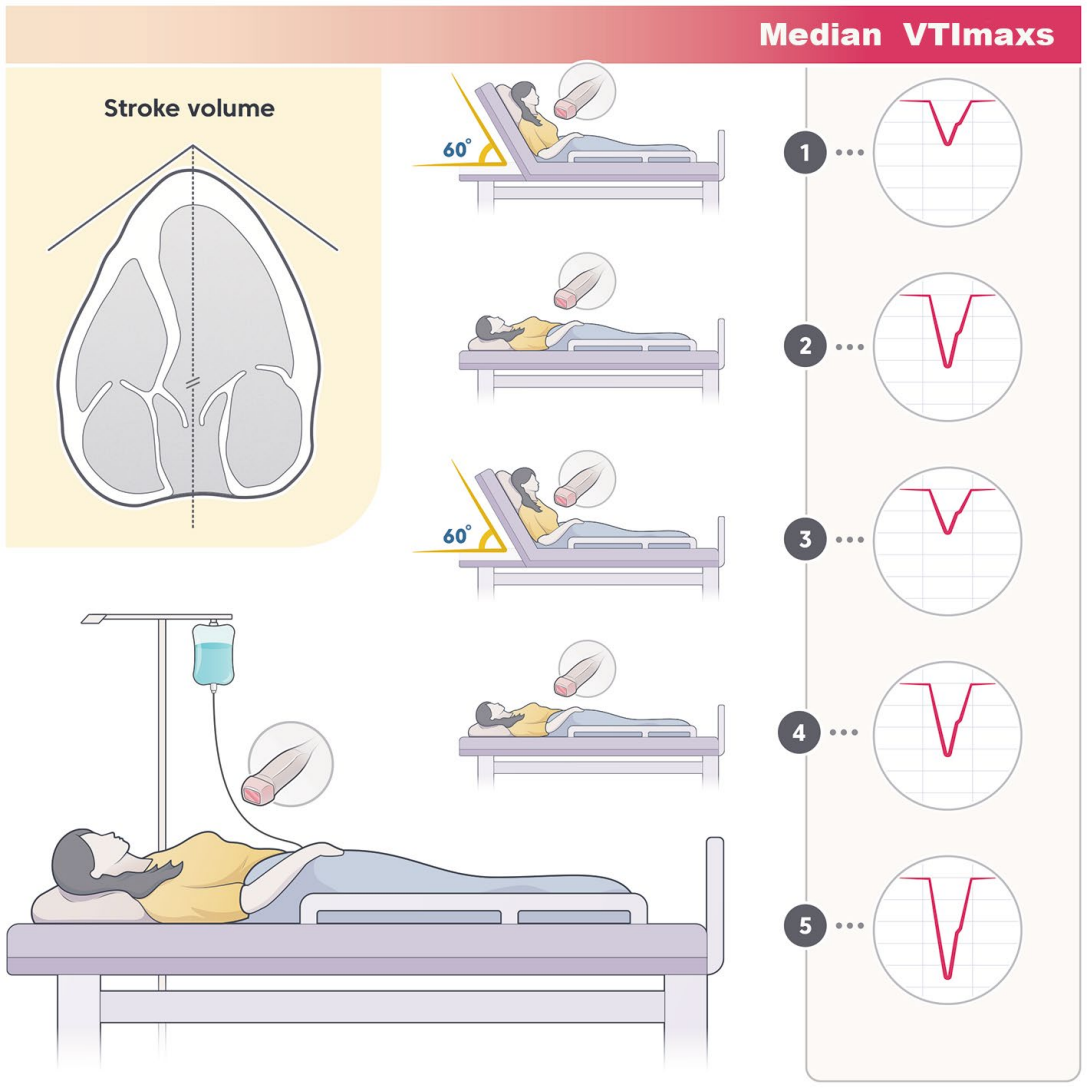
Study procedure using a 60° upper body elevation and a supine position for measuring the mean of medians of maximum velocity time integral and hemodynamic parameters. SV changes from 1 to 2 position indicate UBE-induced medians’ change of VTImaxs; SV changes from 4 to 5 position indicate fluid-induced medians’ change of VTImaxs.

To enhance the accuracy of SV measurement and fluid responsiveness assessment, we manipulated four factors, supported by evidence. First, VTImax, representing SV, was reconfirmed by repeated measurements in the same position to address the potential for uncertain measurements caused by respiratory variations (Fig. 1). Repeated measurements have been shown to improve the precision of SV measurements^15^. Second, we used not all SV values by respiratory variation, but specifically maximum SV values measured during end-expiration. Lower VTI values, other than VTImax, were deliberately excluded after observing the respiratory variation of VTIs, as recommended in a study^15^. Previous studies commonly utilized arbitrary VTIs by respiratory variation over five consecutive cardiac cycles. However, minimum VTI (VTImin) by respiratory variation can be inaccurately measured due to factors such as chest wall movement, subtle examiner hand movements, and cardiac activity itself. These factors can misalign the pulsed-wave Doppler (PW) placement, causing it to shift toward the septal wall and mitral valve (mediolateral directions) and away from the aortic valve (anteroposterior directions), leading to inaccurately low VTImin values and consequently skewing the overall VTI calculations. Unlike VTImin, VTImax is less susceptible to such measurement errors, making it a more reliable metric for assessing fluid responsiveness. VTImax can also be diminished when the location of the PW shifts. However, inaccurately measured VTImax is not considered to represent the true maximum. This VTImax denotes the values recorded during the end-expiration in several cycles and the precision of this measurement method has been proven^15^. Third, we used median VTImax values to ensure that the pulsed-wave (PW) Doppler placement in the anteroposterior direction was at an appropriate distance from the aortic valve. The highest and lowest VTImax values were excluded, as they reflected instances where the PW placement was either too close to or too far from the aortic valve. Lastly, our study eliminated the isolated leg raising stage commonly used in PLR maneuvers, as it showed only transient and minimal effects on SV in previous studies^7,10^. Instead, we opted for a 60° upper body elevation (Fig. 1), supported by evidence that this position induces more significant SV changes^10,16^. In a study, 70° upper body elevation effectively predicted fluid responsiveness^17^. Such a higher angle of upper body elevation may augment the accuracy of SV changes quantification, thereby enhancing the overall validity of fluid responsiveness evaluations.

### SV changes according to position changes in healthy volunteers: criteria to suspect mild hypovolemia

As a preliminary investigation, 54 healthy, spontaneously breathing volunteers over 18 years old and well-hydrated were enrolled. They had adequate nutrition and were free of dehydration symptoms. Exclusion criteria included anatomical limitations and potential dehydration due to poor oral intake or excessive alcohol consumption. Hemodynamic parameters were measured, and fluid challenge tests were conducted on 45 healthy volunteers who fulfilled these criteria. SV changes according to an UBE (UBE-induced medians’ change of VTImaxs, SV changes from the 1st to 2nd position in Fig. 1) and SV changes after fluid loading (fluid-induced medians’ change of VTImax, SV changes from the 4th to 5th position in Fig. 1) were investigated in these volunteers. None of the healthy volunteers exhibited UBE-induced median VTImax change or fluid-induced median VTImax change of 7% or greater, calculated by the mean of median values of VTImaxs. Unlike in hemodynamic unstable patients, the criteria for fluid responsiveness in hemodynamic stable patients or those with mild hypovolemia, have not been thoroughly validated through studies and may be lower as the Frank-Starling curve exhibits a relationship between the degree of hypovolemia and SV^18^. The criteria of fluid responsiveness may differ between normotension^19,20^ and hypotension^21^, and depend on the VTI measurement method used, as in our case using median VTImax change. Fluid responsiveness was defined as a fluid-induced median VTImax change of 10% or greater because fluid responsiveness was lower in mild hypovolemia than in severe hypovolemia based on the Frank-Starling curve. Furthermore, based on the median VTImax change range conducted to determine a criterion of fluid responsiveness as a new measurement method in healthy volunteers, an UBE-induced or fluid-induced median VTImax change of 7% or greater was suspected of mild hypovolemia. The 7% criterion may be due to not using mean values of 5 VTImaxs but using mean values of 5 median VTImaxs.

### SV changes according to position changes in ICU patients

**Figure 2:**
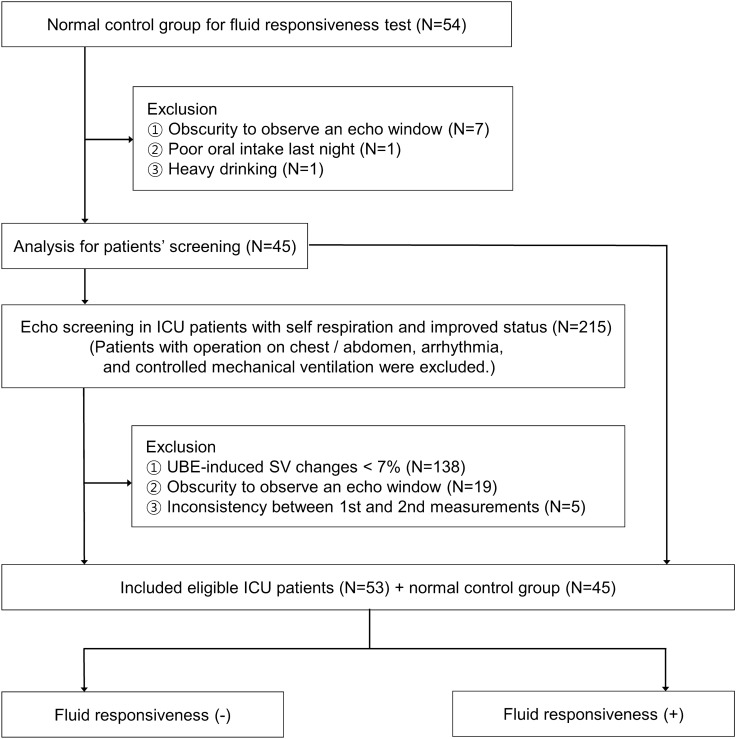
Flow sheet.

Using this normative dataset as a reference, identical hemodynamic parameters were subsequently measured in an ICU patient cohort (Fig. 2). The screening criterion of UBE-induced median VTImax change ≥ 7% was employed to determine eligibility for saline infusion and fluid challenge testing in ICU patients. This approach was chosen to avoid the potential medical and ethical concerns associated with indiscriminate fluid administration. Spontaneously breathing ICU patients who were permitted to receive slight opioids or sedatives, but not vasoactive agents in the recovery phase were screened. Patients on supportive, but not controlled, ventilation were eligible for inclusion. Patients who had too irregular, deep, or shallow respiration were excluded. Additionally, hemodynamic stability in screened ICU patients who showed mild ill looking appearance required: maintaining a MAP of at least 65 mmHg, a urine output of > 0.5 mL/kg per hour, and a normal sinus rhythm (60/min < heart rate < 100/min). Patients with surgical wounds on the abdomen or chest were excluded. Ultrasonographic evaluations were performed on 215 ICU patients who exhibited spontaneous breathing, showed improved clinical status, and were suspected of having mild hypovolemia (mild general weakness or poor oral intake). Of these, 162 were excluded for various reasons: 138 patients had UBE-induced median VTImax change of less than 7%, 19 had an unclear ultrasound window, and 5 displayed inconsistencies between their first and second VTImax measurements in the same position. Ultimately, data from 98 patients who underwent fluid challenge tests, across both healthy volunteers and ICU patients, were included in the final analysis (Fig. 2). Eventually, of 98 subjects, 83 fluid non-responders with fluid responsiveness (fluid-induced median VTImax change ≥ 10%, calculated by the mean of medians of VTImaxs) and 15 fluid responders were compared. Additionally, 63 subjects with fluid-induced median VTImax change < 7% and 35 subjects with fluid-induced median VTImax change ≥ 7% were analyzed.

### Measurement of hemodynamic parameters according to position changes

Before ultrasonographic evaluation, blood pressure was manually measured in healthy volunteers and assessed automatically via an automated cuff in ICU patients. Hemodynamic parameters, including changes in median VTImax and IJV dimensions, were acquired using the ACUSON X300 Ultrasound System (Siemens Medical Solutions USA). The left ventricular outflow tract (LVOT) VTI was manually gauged using pulsed Doppler. To ensure the accuracy of measurements, between 9 and 17 readings for VTImax and peak velocity of VTImax (PV) during end-expiratory respiration, were taken in each position. The mean of the most representative 5 median values was then calculated after discarding the respective highest and lowest 2 to 6 values. These matrics were used to calculate changes in SV and PV as (median VTImax or PVmax in supine—median VTImax or PVmax in UBE)/(median VTImax or PVmax in supine × 1/2 + median VTImax or PVmax in UBE × 1/2). Additionally, the increase ratio in mean VTImax was calculated using a simple formula: median VTImax in supine/median VTImax in UBE. All hemodynamic parameters were re-evaluated at an UBE and in a supine position, followed by subsequently measurements after fluid loading with a volume of 500–700 cc of normal saline. IJV metrics were captured at the level of the cricoid cartilage, recording both the vertical and horizontal diameters as well as the area of the right IJV. These metrics were also used to calculate IJV collapsibility as (IJVmax—IJVmin)/(IJVmax × 1/2 + IJVmin × 1/2) and IJV changes as (IJVmax in UBE—IJVmax in supine) / (IJVmax in UBE × 1/2 + IJVmax in supine × 1/2). To maintain methodological consistency, a single certified echocardiography professional measured all VTImaxs and other hemodynamic parameters. One assistant monitored vital parameters and administered fluid loading, while another managed patient positioning and data recording during measurements.

### Statistical analysis

Continuous variables were presented as medians with interquartile ranges (IQRs), and analyzed using the Mann–Whitney U test. Categorical variables were compared using Fisher’s exact test. To examine the relationship between significant parameters and fluid responsiveness, binary logistic regression was employed. To identify the correlation between VTImax changes after UBE and fluid loading, the correlation coefficient in a linear regression analysis was calculated. The effectiveness of hemodynamic indicators in assessing fluid responsiveness was evaluated using area under the receiver operating characteristic curve (AUC) analysis. All statistical analyses were performed using SPSS v.26 software for Windows (IBM Corp., Armonk, NY, USA).

## Results

**Figure 3:**
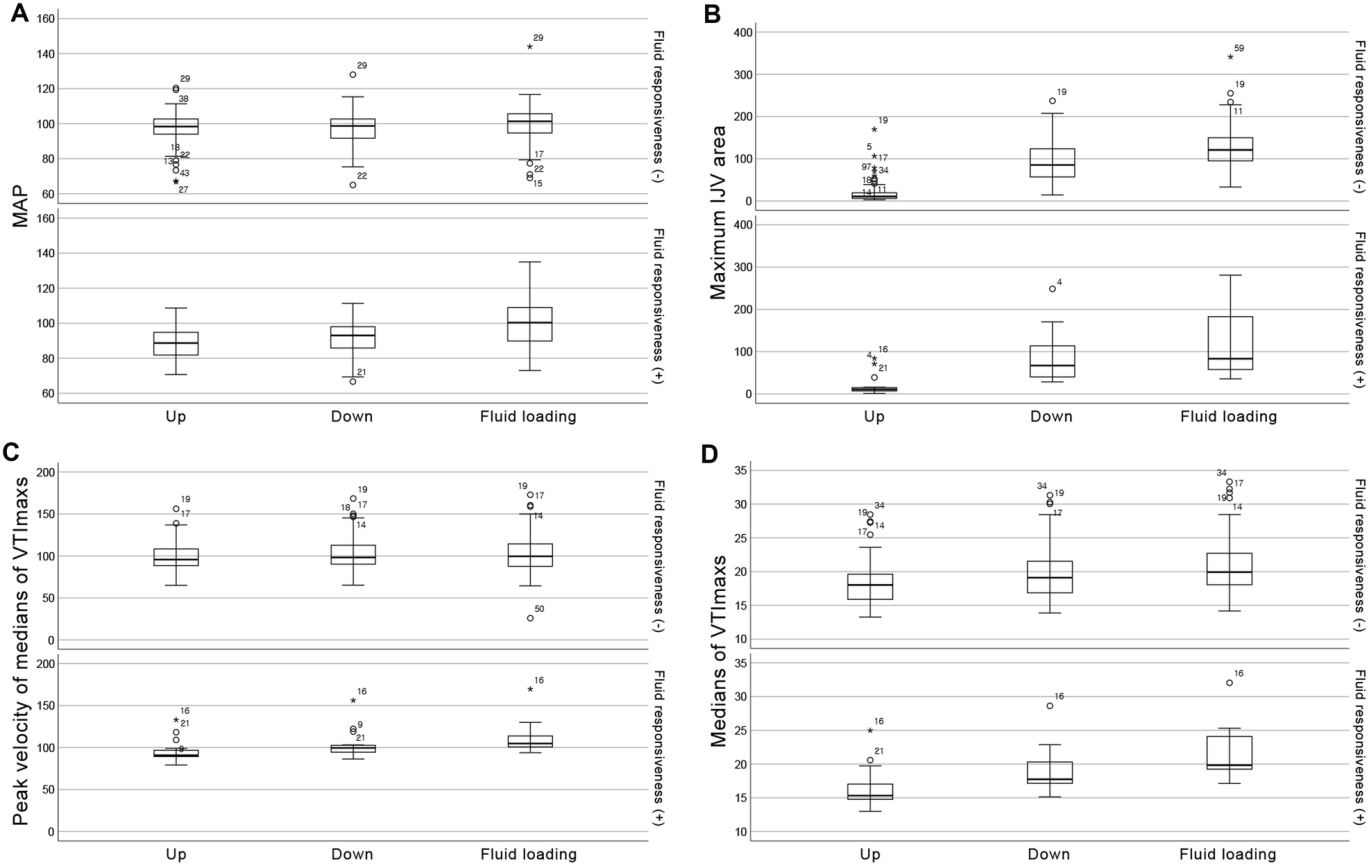
Changes in hemodynamic parameters according position changes and fluid loading in subjects without/with fluid responsiveness. Up denotes 60° upper body elevation; Down, supine; and fluid loading, after fluid loading. (**A**) Mean arterial pressure (mmHg) (**B**) Internal jugular vein collapsibility (%) (**C**) Peak velocity of velocity time integral (cm/s) (**D**) Maximum velocity time integral (cm).

**Figure 4:**
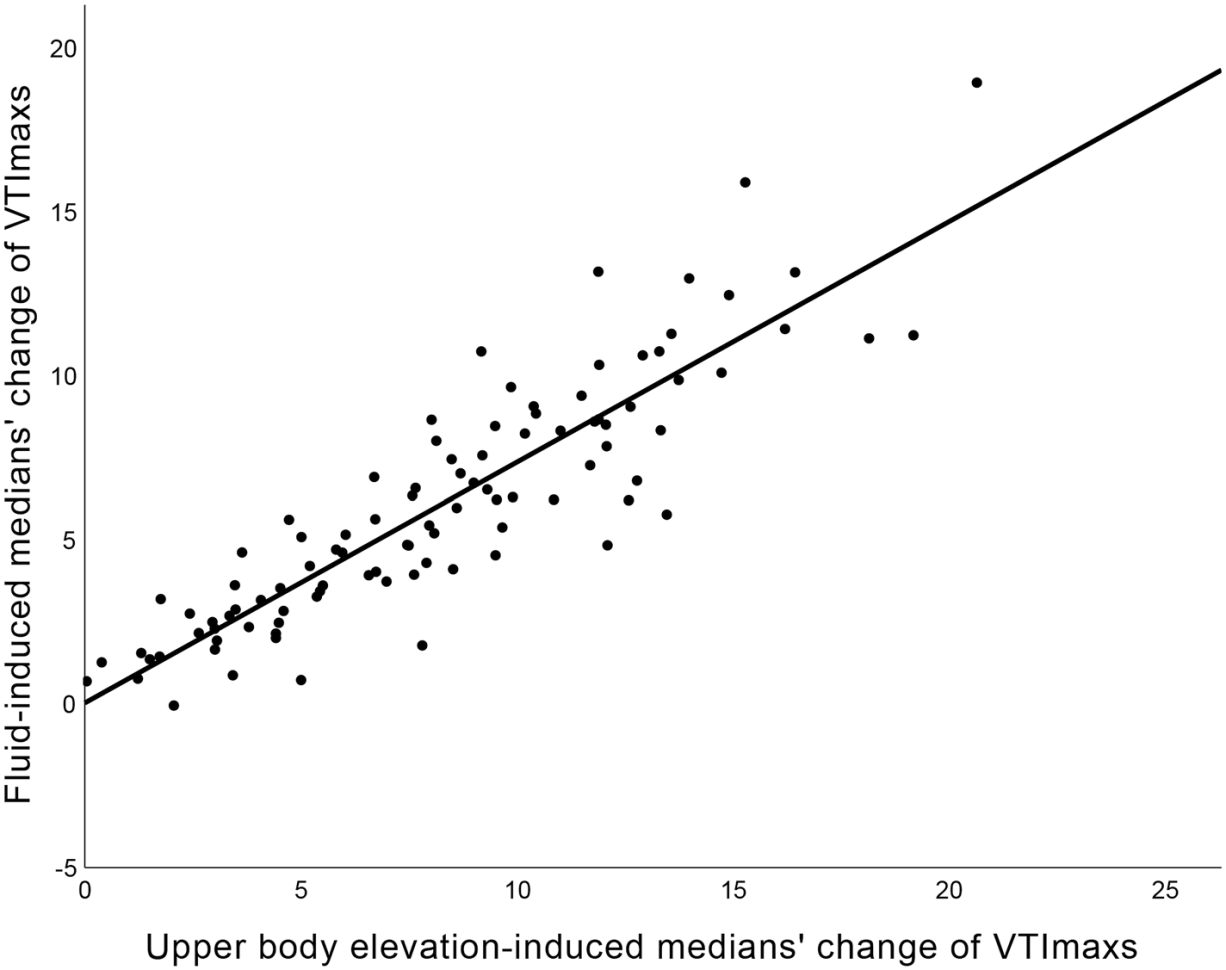
Correlation curve between fluid-induced medians’ change of VTImaxs and 60° upper body elevation-induced medians’ change of VTImaxs.

Of 54 healthy volunteers, 45 were eligible and underwent fluid challenge tests. Of 215 ICU patients who received echocardiographic screening, 53 were eligible and underwent fluid challenge tests (Fig. 2). In the healthy volunteers and ICU patients, the values of hemodynamic parameters were presented in Table 1. Baseline characteristics revealed differences between the responders and non-responders. The median age for the responder group was 77 years, compared to 56 years for the non-responder group. Age was found to be statistically significant (*p* = 0.018), but neither male gender nor body mass index were correlated with fluid responsiveness (fluid-induced median VTImax change ≥ 10%) (*p* = 0.767 and 0.622, respectively). MAP in an UBE position showed a significant decrease for subjects with fluid responsiveness compared to those without (Table 2) (Fig. 3). Median VTImax in an UBE was associated with fluid responsiveness; however, VTImax and PV themselves other than median VTImax in an UBE, did not correlate with fluid responsiveness, although they decreased substantially in the volume responders (Fig. 3). IJVA collapsibility and changes were not associated with fluid responsiveness (Table 2). Multivariable analysis, adjusted for age, identified UBE-induced median VTImax change as the best predictor of fluid responsiveness (*p* = 0.001, odds ratio 90.128) (Table 2) and when fluid-induced median VTImax change was ≥ 7%, the odds ratio was 83.012 (*p* < 0.001). The UBE-induced median VTImax change for predicting fluid responsiveness showed a high-performance level (cut-off value 11.8%, AUC 0.958) and when fluid-induced median VTImax change was ≥ 7%, the performance was AUC 0.936 (cut-off value 7.98%). The relationship between UBE-induced and fluid-induced median VTImax changes was moderately correlated (R^2^ = 0.793, *p* < 0.001) (Fig. 4). For simple calculation, increases ratio greater than 11.2% and 7.7% in median VTImax change when transitioning from a 60° upper body elevation to a supine position effectively predicted fluid responsiveness and fluid-induced median VTImax change ≥ 7% (AUC 0.958 and 0.936).

**Table 1:** Changes in ultrasonographic parameters according to position changes and fluid loading.

	Up position		Down position		SVCP or IJVC (%)	Fluid loading	SVCF or IJVC (%)
	1st	2nd	1st	2nd			
Normal control group							
Mean PVmax, cm/s	93 (83.1, 100.1)	90.7 (82.9, 99.2)	93.2 (84.8, 101.3)	92.6 (83.3, 101.1)		90.6 (81.8, 99.7)	
Mean VTImax, cm	16.9 (15.3, 18.4)	16.7 (15.3, 18.4)	18 (16, 19.1)	18 (15.9, 19.2)	4.1 (3.1, 6)	18.5 (16.5, 20)	2.88 (1.85, 4.25)
IJVDmax, mm	2.6 (1.8, 3.2)		8.4 (6.9, 10.5))		Up: 13 (8.8, 19.3)	10.1 (9.2, 11.5)	8.8 (4.6, 11.6)
IJVDmin, mm	2.3 (1.5, 2.7)		7.4 (5.9, 9.2)		Down: 13.6 (8.7, 20.5)	9.2 (8, 10.6)	
IJVAmax, mm^2^	9.2 (5.9, 12.5)		83.6 (62.8, 118.7)		Up: 26.4 (13.1, 35.5)	126.7 (98.6, 151.6)	15.5 (10.4, 19.4)
IJVAmin, mm^2^	7.5 (4.2, 10.7)		68.8 (44.6, 99.7)		Down: 25.7 (16.6, 33.1)	105.1 (81.3, 129.5)	
ICU patient group (SVCP ≥ 7% indicated suspected mild hypovolemia; volume responsiveness was defined as SVCF ≥ 10%)							
Mean PVmax, cm/s	101.1 (89.4, 114)	100.4 (89.5, 113.4)	104.4 (96.5, 120.4)	104.2 (96, 121.3)		109.8 (100.7, 129)	
Mean VTImax, cm	18.7 (15.3, 20.6)	18.8 (15.3, 20.5)	20.7 (17.4, 23.1)	21.3 (17.9, 23.7)	10.8 (9, 13.3)	22.3 (19.5, 25.3)	8.3 (6.3, 10.5)
IJVDmax, mm	3.3 (2.3, 5.3)		7.4 (5.6, 10.5)		Up: 20.9 (11.4, 33)	9.5 (7.4, 12.1)	14.3 (5.3, 21.8)
IJVDmin, mm	2.6 (1.7, 3.6)		5.7 (3.9, 8.7)		Down: 20.7 (6.1, 37.9)	8 (5.5, 10.8)	
IJVAmax, mm^2^	13.5 (6.8, 28.6)		72.6 (40.6, 127.9)		Up: 36.2 (21.5, 68.5)	118.3 (66.6, 165.5)	20.6 (14.2, 37.2)
IJVAmin, mm^2^	9.3 (4.1, 15.9)		46.7 (29.4, 91.5)		Down: 27.1 (11.5, 63.8)	80.2 (43, 128.8)	

Up position denotes 60° upper body elevation; Down position, Supine; SVCP, stroke volume changes after position change; SVCF, stroke volume changes after fluid loading; IJVC, internal jugular vein collapsibility; PV, peak velocity of the stroke volume; VTI, velocity time integral; IJVD, vertical diameter of the internal jugular vein; IJVA, area of the internal jugular vein.

**Table 2:** Hemodynamic parameters to predict fluid responsiveness.

Univariable	Fluid responsiveness (−)	Fluid responsiveness (+)	*p*	AUC
Systolic blood pressure, Up	135 (130, 142)	126 (102, 140)	0.068	0.351
Systolic blood pressure, Down	135.5 (128, 142)	131 (106, 153)	0.273	0.41
Systolic blood pressure changes	0 (− 3.7, 3.1)	4.2 (− 3.7, 7.7)	0.068	0.649
Diastolic blood pressure, Up	80 (72, 84)	70 (66, 75)	0.01	0.291
Diastolic blood pressure, Down	79.5 (75, 84)	73 (67, 82)	0.08	0.357
Diastolic blood pressure changes	1.2 (− 4.6, 6.2)	4.2 (− 3.7, 7.7)	0.337	0.578
Mean arterial pressure, Up	98.3 (94, 102.7)	88.7 (80.7, 97.7)	0.004	0.268
Mean arterial pressure, Down	98.7 (91.6, 102.8)	93 (84, 98.7)	0.055	0.343
Mean arterial pressure changes	0.2 (− 3.6, 4.3)	4 (− 1.5, 9.2)	0.121	0.627
Peak velocity, Up	95.7 (88.3, 108.6)	90.8 (89.1, 99)	0.441	0.437
Peak velocity, Down	98.2 (90.1, 113.1)	99.6 (94.2, 103.1)	0.676	0.534
Mean peak velocity changes	3.2 (0.5, 6)	8.7 (4, 10.5)	< 0.001	0.804
Velocity time integral, Up	18 (15.7, 19.7)	15.3 (14.4, 18.5)	0.023	0.314
Velocity time integral, Down	19.1 (16.6, 21.6)	17.7 (17, 21.5)	0.307	0.417
Mean stroke volume changes	7.5 (4.1, 9.7)	14.7 (12.9, 16.4)	< 0.001	0.959
IJVD collapsibility, vertical, Up	16.1 (10.9, 24.5)	15.4 (8.8, 30.5)	0.862	0.514
IJVD collapsibility, vertical, Down	16.7 (8.1, 25.4)	14.8 (5.3, 34.9)	0.846	0.516
IJVD vertical changes	90.6 (63.3, 115.9)	87.7 (51.1, 123.3)	0.783	0.478
IJVA collapsibility, Up	28.3 (18.3, 47.8)	35.8 (27.4, 89)	0.096	0.636
IJVA collapsibility, Down	26.1 (14.9, 40)	15.7 (7.7, 57)	0.668	0.465
IJVA changes	146.2 (115, 167)	128.3 (106.4, 170.1)	0.481	0.443

Multivariable	Coefficient	95% CI	*p*	OR
Mean arterial pressure, Up	− 0.577	0.241–1.308	0.181	0.562
Mean stroke volume changes	4.501	5.686–1428.529	0.001	90.128
IJVA collapsibility, Up	0.492	0.703–3.801	0.253	1.635

## Discussion

Methods for accurately measuring SV and its changes, such as repeated measurements, VTImax during end-expiration, medians of VTImax values, and 60° upper body elevation, were employed to evaluate fluid responsiveness in hemodynamically stable patients not requiring mandatory ventilation or sedation, utilizing fluid challenge tests. In the healthy volunteers, there were no instances of UBE-induced median VTImax change or fluid-induced median VTImax change ≥ 7%. Utilizing UBE-induced median VTImax change enabled the screening of mild hypovolemia and the prediction of fluid responsiveness, which was determined by fluid-induced median VTImax change ≥ 10%, calculated by the mean of the medians of VTImax values. Our findings suggest that fluid responsiveness can be accurately predicted using UBE-induced median VTImax change in spontaneously breathing patients with hemodynamic stability.

Measuring precise SV in hemodynamically stable subjects with spontaneous breathing is extremely challenging, although accurate measurements of VTIs are crucial for differentiating fluid responsiveness. We measured VTImax in the expiratory phase, excluding VTImin in the inspiratory phase. A reliable study showed that repeated measurements of VTImax are a precise method, and the accuracy of VTImax measurements depends on the number of measurements^15^. It is essential to measure the median VTImax change recorded during end-expiration and to average three or more VTImax measurements for precise SV measurement using transthoracic echocardiography^15^. Additionally, after excluding VTImin values, we discarded the respective highest and lowest 2 to 6 from a total of 9 to 17 readings of VTImax, as LVOT VTI should ideally be measured 1 cm from the aortic valve; however, the anterioposterior movement of the PW, due to cardiac motion, can lead to imprecise VTImax values if those are measured too close to or far from the aortic valve. The reason for the low cut-off value of SV changes for suspecting mild hypovolemia in our study needs explanation although deducted from the results in normal control group. Firstly, if a study includes a high proportion of the population with mild hypovolemia or hemodynamic stability, SV changes may be small, as shown in the Frank-Starling curve. In this cohort, the criterion for fluid responsiveness may be an SV change ≥ 10%. In a study where SV was measured using esophageal Doppler, none of the healthy volunteers showed an increase in SV ≥ 10% following fluid administration, and normovolemia was defined as SV changes ≤ 10%^22^. The cut-off value of SV changes might be lower because the SV changes after fluid loading were not over 7% in our normal control group. The lower cut-off value for predicting mild hypovolemia may be due to the selection of median VTImax change in our study. Discarding the respective highest and lowest 2 to 6 values of 9 to 17 readings of VTImax according to cardiac cycles for precise measurements may result in lower SV changes. In terms of precision, the criterion using median VTImax values in our study demonstrated higher performance for predicting fluid responsiveness in hemodynamically stable subjects with spontaneous breathing.

For precise SV measurements, another method employed was 60° upper body elevation alone, excluding the use of isolated leg raising. In healthy subjects, several studies have explored the impact of body positioning on aortic SV indexes. One study measured SV indexes in sitting, semi-recumbent, supine positions, and at 60° lower body elevation, respectively. The changes in these values corresponded to a 36% increase in the semi-recumbent position and a 3.2% increase at 60° lower body elevation^10^. Another study found SV values of 81.1 mL and 90.7 mL at 60° and 30° upper body elevations, respectively, with a larger decrease in SV at higher upper body elevations^23^. Furthermore, a study comparing SVs in supine and 45° lower body elevation positions in healthy subjects found SV values of 69 and 74 mL, indicating a 7% change^24^. The utility of isolated leg elevation for assessing fluid responsiveness might exist; however, compared to 60° upper body elevation, the change in SV using isolated leg elevation appears minimal under normal physiological conditions. Moreover, the hemodynamic effects of isolated leg elevation were observed to dissipate after 7 min^9^. Given that the hemodynamic effect of isolated leg elevation on SV decreases over time and that the magnitude of change is relatively small, the isolated leg raising procedure might be redundant for accurately measuring SV in hemodynamic stable patients. Several studies have explored the efficacy of upper body elevation tests, excluding lower body elevation, in assessing fluid responsiveness. One study focusing on mechanically ventilated patients discovered a significant correlation between fluid responsiveness and a change in pulse pressure variation greater than 8% when patients were transitioned from a supine position to a 90° sitting position^25^. Another investigation noted that an increase in SVV of more than 12% when moving from supine to 70° upper body elevation effectively predicted fluid responsiveness^17^. Such findings suggest that upper body elevation tests may offer a convenient and reliable method for assessing fluid responsiveness across various clinical settings. The accuracy of current PLR test might be enhanced by adopting a steeper upper body elevation angle and forgoing the isolated leg raising procedure to measure SV changes more precisely. However, angles of 60° or steeper might result in discomfort.

Maintaining a consistent tidal volume is critical. In sedated and mechanically ventilated patients, an increase in tidal volume correlates with an increased SVV^26^. Similarly, in spontaneously breathing patients, shallow or deep breathing significantly affects SVV. Studies have shown that employing controlled ventilation in these patients leads to more precise SVV measurements^12,13^. If breathing is unusually shallow or deep, volume assessment criteria become less precise. Our study did not include subjects with breathing issues. For a reliable volume assessment, it is imperative to maintain consistent and optimal tidal volumes, avoiding irregular or excessive breathing patterns.

Several previous studies have supported the utility of IJV distensibility in accurately predicting fluid responsiveness in mechanically ventilated septic patients^27,28^. Additionally, a study indicated that IJV collapsibility could reliably predict hypovolemia in patients with spontaneous breathing^29^. However, contrasting findings have emerged, suggesting that IJV collapsibility may not effectively predict fluid responsiveness in spontaneously breathing patients, as indicated by our results^30^. This inconsistency might be due to the influence of thoracic or intra-abdominal pressures on the IJV during spontaneous breathing, which could compromise measurement accuracy^5^.

This study has several limitations. First, there was a notable age difference between the responders and non-responders. Established findings suggest that the SV decrease in healthy young subjects when the upper body is elevated to 60° is more pronounced than in older subjects^16^. Although we adjusted for age as a covariate, fully mitigating the influence of age is challenging. The younger age of the fluid non-responders might result in more substantial SV changes, but this likely has minimal impact on evaluating fluid responsiveness. The higher cut-off value of SV changes observed in younger fluid non-responders may not be a significant concern, as it only slightly elevates the screening threshold for SV changes in older fluid responders. Second, patients’ respiration was not controlled during the study. Controlled respiration typically yields more precise results, and slow-patterned breathing may enhance the discriminative ability of SVV by increasing baseline SV^31^. Third, our findings may be less applicable in clinical scenarios characterized by low lung compliance, increased pulmonary artery pressure, cardiac arrhythmias, intra-abdominal hypertension, or mandatory ventilation with extreme tidal volumes. These conditions could affect the cut-off value for SV change when assessing fluid responsiveness^32^. Lastly, the novel method for measuring changes in SV should be compared with the traditional SVV measurement techniques and rigorously validated from multiple perspectives. The passive leg raising method and the upper body elevation method have not been directly compared, and this new measurement approach has not been studied in hemodynamically unstable patients. In this study, the method for measuring VTImax at end expiration should be compared with the conventional consecutive VTI measurement. Further supportive studies are required to demonstrate the superiority of this methodology.

In conclusion, UBE, without leg raising, and the mean change in medians of VTImax values proved to be reliable screening tools for suspecting mild hypovolemia and assessing fluid responsiveness in spontaneously breathing patients maintaining hemodynamic stability. The screening tool (UBE-induced median VTImax change ≥ 11.8%) was significantly correlated with fluid responsiveness (fluid-induced median VTImax change ≥ 10%, calculated by the mean of medians of VTImax values); however, the collapsibility of the IJV was not reliable. Given the limitations and scope of this study, future research is warranted to validate these findings. Additional studies employing the same method are essential to confirm the utility and reliability of using UBE and the mean change in medians of VTImax values during end-expiration for assessing fluid responsiveness in a broader spectrum of clinical settings.

## Data Availability

The datasets analyzed during the current study are available from the corresponding author on reasonable request.
